# Jasmonate-Induced Defense Mechanisms in the Belowground Antagonistic Interaction Between *Pythium arrhenomanes* and *Meloidogyne graminicola* in Rice

**DOI:** 10.3389/fpls.2019.01515

**Published:** 2019-11-22

**Authors:** Ruben E. M. Verbeek, Evelien Van Buyten, Md Zahangir Alam, David De Vleesschauwer, Jonas Van Bockhaven, Takayuki Asano, Shoshi Kikuchi, Ashley Haeck, Kristof Demeestere, Godelieve Gheysen, Monica Höfte, Tina Kyndt

**Affiliations:** ^1^Department of Biotechnology, Faculty of Bioscience Engineering, Ghent University, Ghent, Belgium; ^2^Laboratory of Phytopathology, Department of Plants and Crops, Faculty of Bioscience Engineering, Ghent University, Ghent, Belgium; ^3^Plant Genome Research Unit, Agrogenomics Research Center, National Institute of Agrobiological Sciences, Tsukuba, Japan; ^4^Research Group EnVOC, Department of Sustainable Organic Chemistry and Technology, Ghent University, Ghent, Belgium

**Keywords:** rice, oomycete, root-knot nematode (*Meloidogyne graminicola*), jasmonate, auxin, antagonism

## Abstract

Next to their essential roles in plant growth and development, phytohormones play a central role in plant immunity against pathogens. In this study we studied the previously reported antagonism between the plant-pathogenic oomycete *Pythium arrhenomanes* and the root-knot nematode *Meloidogyne graminicola*, two root pathogens that co-occur in aerobic rice fields. In this manuscript, we investigated if the antagonism is related to imbalances in plant hormone levels, which could be involved in activation of plant defense. Hormone measurements and gene expression analyses showed that the jasmonate (JA) pathway is induced early upon *P. arrhenomanes* infection. Exogenous application of methyl-jasmonate (MeJA) on the plant confirmed that JA is needed for basal defense against both *P. arrhenomanes* and *M. graminicola* in rice. Whereas *M. graminicola* suppresses root JA levels to increase host susceptibility, *Pythium* inoculation boosts JA in a manner that prohibits JA repression by the nematode in double-inoculated plants. Exogenous MeJA supply phenocopied the defense-inducing capacity of *Pythium* against the root-knot nematode, whereas the antagonism was weakened in JA-insensitive mutants. Transcriptome analysis confirmed upregulation of JA biosynthesis and signaling genes upon *P. arrhenomanes* infection, and additionally revealed induction of genes involved in biosynthesis of diterpenoid phytoalexins, consistent with strong activation of the gene encoding the JA-inducible transcriptional regulator DITERPENOID PHYTOALEXIN FACTOR. Altogether, the here-reported data indicate an important role for JA-induced defense mechanisms in this antagonistic interaction. Next to that, our results provide evidence for induced expression of genes encoding ERF83, and related PR proteins, as well as auxin depletion in *P. arrhenomanes* infected rice roots, which potentially further contribute to the reduced nematode susceptibility seen in double-infected plants.

## Introduction

Rice (*Oryza sativa*) is an important staple crop for a large part of the world population. Moreover, researchers use this plant as model organism for monocots. Sessile organisms, such as rice and other plants, have evolved sophisticated strategies to fend off pathogens. Phytohormones play a central role in the orchestration of plant immunity against pathogens and jasmonic acid (JA), salicylic acid (SA), and ethylene (ET) are considered the backbone of this network. In addition, abscisic acid (ABA), auxins (AUX), gibberellins, cytokinins, and brassinosteroids have also emerged as players in plant defense (Pieterse et al., 2012; De Vleesschauwer et al., 2013).

Jasmonates are lipid-derived signal molecules that play vital roles in diverse physiological processes, but have mostly been studied for their involvement in wounding responses, secondary metabolite biosynthesis, and defense. JA is synthesized through the oxylipin biosynthesis pathway starting from α-linolenic acid from membrane lipids. JA can further be metabolized to the volatile methyl-jasmonate (MeJA), or conjugated with amino acids such as Ile to the biologically active JA-Ile compound through the enzyme JA-Ile synthase (JAR1) (Pieterse et al., 2012; Lyons et al., 2013). JA-Ile plays a vital role in jasmonate signaling by activating genes that are suppressed by transcriptional repressor proteins with a Jasmonate ZIM (JAZ) domain. In the absence of JA, JAZ proteins bind to transcription factors (TFs) of JA-responsive genes and prevent their expression. The F-box protein Coronatine-Insensitive1 (COI1), a key regulator in the activation of JA-responsive genes, functions as the receptor of JA-Ile as part of the E3 ubiquitin-ligase SKP1-Cullin-F-box SCF^COI1^complex. Upon binding to JA-Ile, this complex directs ubiquitination and subsequent degradation of JAZ proteins *via* the proteasome (Pauwels and Goossens, 2011; Pieterse et al., 2012), leading to activation of JA-response genes. In *Arabidopsis*, the JA-responsive genes can be divided in two branches, where the MYC genes are associated with wound response and defense against insect herbivores, while other genes known as APETALA2/Ethylene Response Factor (AP2/ERF) genes are controlled by both JA and ET and are associated with enhanced resistance to necrotrophic pathogens (Zhu et al., 2011).

Although very relevant for agronomy, pathogenic interactions between plants and multiple damage-causing organisms are largely understudied, certainly when considering the role of plant hormones and plant defense. Two such soil-borne pathogenic organisms that co-occur in aerobic rice fields are *Pythium arrhenomanes* and *Meloidogyne graminicola* (Kreye et al., 2009a; Verbeek et al., 2016). The soil-borne oomycete *P. arrhenomanes* infects several monocots such as rice (Kreye et al., 2009a), sugarcane (Bond et al., 2004), and wheat (Mojdehi et al., 1991). In a field study, *P. arrhenomanes* was found to be the most virulent species isolated from aerobic rice fields (Van Buyten et al., 2013). *P. arrhenomanes* can cause seed mortality, damping off, and stunting of young seedlings (Kreye et al., 2009b). The role of phytohormones in plant defense against *P. arrhenomanes* is poorly understood. However, in the *Pythium graminicola*-rice interaction, the oomycete has been reported to hijack the brassinosteroid pathway to antagonize gibberellin and SA-dependent defense pathways (De Vleesschauwer et al., 2012). ET signaling plays a positive role in pea immunity against *Pythium irregulare* (Blake et al., 2015), since ET insensitive *ein2*-mutant pea plants were shown to be highly susceptible. In the moss *Physcomitrella patens*, infection by *P. irregulare* and *Pythium debaryanum* activates expression of the defense genes chalcone synthase, lipoxygenase, and phenylalanine ammonia lysase, as well as accumulation of reactive oxygen species, JA, and its precursor 12-oxo-phytodienoic acid (OPDA) (Oliver et al., 2009). In a similar manner, *Pythium ultimum* induces expression of ET and JA biosynthesis and response genes in apple (Shin et al., 2014). In *Arabidopsis*, Staswick et al. (1998) showed that JA signaling is needed for defense against *P. irregulare*.

*M. graminicola* is one of the most damaging pathogens in aerobic rice (Mantelin et al., 2017). This root-knot nematode induces the formation of giant cells inside root vascular tissue of its host plant, leading to the visual symptoms of root galling and aboveground chlorosis (Mantelin et al., 2017). The *M. graminicola*-rice interaction has been studied thoroughly on a molecular level, revealing a major transcriptional repression of host defense pathways, in contrast to activation of cytokinesis and primary metabolic pathways (Kyndt et al., 2012; Ji et al., 2013). JA plays a pivotal role in the basal defense against *M. graminicola* in rice, while exogenous supply of benzothiadiazole (BTH)—a SA-analogue—only has minor defense-inducing capacity (Nahar et al., 2011). Exogenous application of ethephon (an ET-releaser) results in enhanced defense against this nematode, but works in a JA-dependent manner. Hormones which are traditionally known to be important for plant growth and development or abiotic stress responses rather promote host susceptibility to this nematode. For example, recent data show an accumulation of ABA in galls at 3 and 7 days post *M. graminicola* inoculation in rice, and exogenous application of ABA overrules JA-induced defense (Kyndt et al., 2017). In a similar manner, activation of gibberellins, which accumulate in nematode feeding sites, promotes rice root susceptibility while it suppresses the JA pathway (Yimer et al., 2018). AUX accumulation is also of crucial importance for *M. graminicola*-induced feeding site formation in rice (Yimer et al., 2018), as well as in dicotyledonous host plants (Kyndt et al., 2016).

Previously we showed an antagonistic interaction between the plant-pathogenic oomycete *P. arrhenomanes* and *M. graminicola*, where infection with the oomycete negatively affects the number of established nematodes and hampers nematode development in rice roots (Verbeek et al., 2016). We hypothesized that this antagonism could be related to changes in phytohormones and related induced defense responses in the rice roots. The goals of the current study were (i) to investigate how phytohormones SA, JA, AUX, and ABA are affected by single and double inoculation in rice roots, (ii) to investigate which phytohormones play a key role in this antagonistic interaction between both pathogens in rice roots, and (iii) to provide more insights into the transcriptional response of rice roots upon *Pythium* infection.

## Materials and Methods

### Rice Varieties

Rice (*O. sativa*) wild-type varieties used in this study include cultivar “Nipponbare” (provided by the U.S. Department of Agriculture; GSOR-100) and breeding line IR81413-BB-75-4 (provided by the Plant Breeding, Genetics and Biotechnology Division of International Rice Research Institute (IRRI). Transgenic and mutant lines include Tos17-insertion mutant *Osjar1* [NC0364; http://tos.nias.affrc.go.jp/; (Miyao et al., 2003)] and transgenic *OsCOI1-18* RNAi-line (Yang et al., 2012), which both have Nipponbare as background. Seeds were stored at 4°C. Seeds were dehusked, surface sterilized with 4% hypochlorite for 15 min, and subsequently washed three times with sterile water before germination.

### Preparation of *Pythium* Inoculum

*P. arrhenomanes* (isolate PT60), isolated from an aerobic rice field in Tarlac, Philippines (Van Buyten et al., 2013), was maintained in water agar plugs submerged in sterile distilled water and kept at 15°C. Working cultures were revived on potato dextrose agar (PDA) and incubated at 28°C. Final inocula for the greenhouse experiment were prepared by inoculating one-fourth of a 3-day-old PDA plate into a glass jar containing 150 g sterile rice grain:rice hull (RG : RH, 1:3) substrate for 7 days.

### Maintenance of *M. graminicola*

*M. graminicola* was originally isolated in the Philippines (Batangas) and was kindly provided by Dirk DeWaele (Catholic University, Leuven, Belgium). Nematode cultures were maintained *in vivo* on wild-type Nipponbare plants and grasses (*Echinocloa crus-galli*) as described by Nahar et al. (2011). Collection of nematodes was performed on three-months old plants with the Baermann funnel method described by Kyndt et al. (2017). Inoculation conditions are described below per experiment. The nematode infection level was assessed by counting the number of galls and nematodes after visualizing them by boiling whole roots for 3 min in 0.8% acetic acid and 0.013% acid fuchsin. Afterward, the roots were washed with running tap water and slowly destained in acidified glycerol. The developmental stage of each nematode was scored to assess the nematode development and egg masses were counted as an estimate of reproduction.

### *In Vitro P. Arrhenomanes* Infection

Six sterilized rice seeds were placed 2 cm apart on square Petri dishes (120 × 120 mm) filled with 50 ml of Gamborg B5 basal medium with 1% plant agar. Plants were grown at 26°C under a 12-h/12-h light regime. For some experiments, Gamborg B5 basal medium was amended with 25 µM MeJA or 10 µM 5,8,11,14-eicosatetraynoic acid (ETYA). Three days post imbibition, when the primary roots were approximately 1 cm in length, the seeds were infected with *P. arrhenomanes* (PT60).Pythium plugs were pressed out with a cork screw (4#) from a PDA culture (henceforth named PaPDA). Plugs were placed between seedlings 1 and 2, seedlings 3 and 4, and seedlings 5 and 6. At 7 days post inoculation, the plants were measured and the plant health was assessed according to two scoring systems; (i) a shoot disease index (Van Buyten and Höfte, 2013): healthy green shoot (0); yellow spots (1); brown spots (2); dead (3), and (ii) a root disease index (De Vleesschauwer et al., 2012):

- root length >60% of mock treatment, necrosis covering <20% of total root area,
- root length >60% of mock treatment, necrosis covering >20% of total root area,
- root length 20% to 60% of the respective mock treatment,
- root length <20% of mock treatment, necrosis covering <75% of total root area,
- root length <20% of mock treatment, necrosis covering >75% of total root area.

Disease index values were calculated according to the following formula:

$$\mathrm{DIV}=\frac{\left(a+2b+3c+4d+5e\right)}{\left(a+b+c+d+e\right)}$$

### Coinoculation Experiments

#### Greenhouse Experiment

Root samples of variety IR81413-BB-75-4 were collected during the greenhouse experiment performed at the IRRI in Los Baños, Philippines (Verbeek et al., 2016). Detailed experimental conditions were described in Verbeek et al. (2016). Sterilized seeds were germinated in tap water, which was refreshed daily for one week. Seven-day-old seedlings were transplanted in four equally spaced points per pot. One day prior to transplanting, 150 g of rice grain:rice hull (RG : RH) with *P. arrhenomanes* was incorporated in the soil (1:40 ratio) for the *P. arrhenomanes* inoculated pots. The same amount of sterile un-inoculated RG : RH was added to the other pots. Nematode inoculation was performed by adding 750 second-stage juveniles (J2) on each side of the seedlings at 1 day post transplanting (dpt), totaling 6,000 J2s per pot. The same inoculation procedures were followed for the *P. arrhenomanes*-*M. graminicola* combination treatments, using four different treatment groups with 18 plants: (i) un-inoculated control, (ii) *P. arrhenomanes* single inoculation + nematode mock inoculation, (iii) *M. graminicola* single inoculation + oomycete mock inoculation, and (iv) *P. arrhenomanes* + *M. graminicola* double inoculation. At dedicated time points, root samples were taken, flash frozen in liquid nitrogen and lyophilized for transport to Belgium for further analysis.

#### Plant Growth Room Experiments in SAP Substrate

Sterilized rice seeds were germinated for 3 days on wet filter paper at 30°C, and subsequently transferred to Sand and Absorbent Polymer (SAP) substrate placed in specially made polyvinyl-chloride (PVC) tubes (Reversat et al., 1999). Plants were grown at 26°C under a 12-h/12-h light regime. Five day old seedlings were inoculated with two PaPDA plugs by pressing the plugs in the SAP in close proximity of the root. Subsequently the SAP was drenched with 10 ml distilled water to cover the PaPDA plugs. Mock inoculated plants were treated with two empty PDA plugs. Four days later, 250 second-stage juveniles of *M. graminicola* were inoculated per plant (visualized in **Figure S1**). Two treatment groups, each consisting of eight individual plants, were used in these experiments: (i) *M. graminicola* single inoculation + oomycete mock inoculation, and (ii) *P. arrhenomanes* + *M. graminicola* double inoculation. At 12 days post nematode inoculation, nematode infection was evaluated by staining the root systems with acid fuchsin to visualize galls and nematodes.

For MeJA treatment, seedlings were sprayed 24 h prior to nematode inoculation with 100 µM MeJA (Sigma) or water. MeJA was first dissolved in 1 ml of methanol and subsequently diluted in 50 ml distilled water containing 0.02% (v/v) Tween 20. The solution was sprayed with a vaporizer on the shoots until run off. Distilled water containing 0.02% (v/v) Tween 20 was used as a control treatment. All experiments were independently repeated twice, with similar results, with in each case at least six individual plants per treatment.

### SA, JA, IAA, and ABA Measurement

A cold extraction of 100 mg of homogenized rice material (root or shoot) was performed using the modified Bieleski solvent, followed by filtration and clean-up. Chromatographic separation of JA, SA, indole-3-acetic-acid (IAA), and ABA was achieved on an ultra-high performance liquid chromatography system coupled to a Q Exactive™ quadrupole Orbitrap mass spectrometer (Thermo Fisher Scientific). A Nucleodur C18 column (50 × 2 mm; 1.8 µm d_p_) was used, with a mobile phase gradient consisting of acidified methanol and water. Mass spectrometric analysis was carried out in both positive and negative electrospray ionization and in selected-ion monitoring mode, at a mass resolution of 70,000 full width at half maximum. For more details about this method, see Haeck et al. (2018).

### Data Analysis

SPSS (version 23, IBM, USA) was used for all statistical analyses. The normality of the data was checked using the Kolmogorov-Smirnov test of composite normality (α = 0.05). None of the data were normally distributed; therefore, non-parametric Mann-Whitney U test (P ≤ 0.05) was performed. Specific details on the statistical analyses are given per experiment in the respective legends.

### Transcriptome Analysis

Seeds were grown in Gamborg B5 medium as described above, using 0.6-cm PDA plugs of a 7-day-old culture of *P. arrhenomanes* (PT60). Samples from mock-inoculated and *Pythium*-infected seedlings were collected 2 days post inoculation (dpi). Three biological replicates were sampled, where each biological replicate represents a pool from at least 20 individual plants, giving a total of six RNA samples (two treatments × three biological replicates). Total RNA was extracted from frozen root tissue using the spectrum plant total RNA kit (Sigma-Aldrich) and subsequently Turbo DNase treated according to the provided protocol (Ambion). For transcriptome analysis, a two-dye method was used described by (Satoh et al., 2010), which will allow direct comparison between two samples and decreases technical bias. A total of 850 ng of RNA was synthesized to cyanine 3- or cyanine 5-labeled cDNA using a low-input RNA labeling kit (Agilent Technologies) and hybridized to custom-made 60-mer Agilent arrays according to the manufacturer’s protocols (Agilent Technologies). Slide image files were generated using a DNA microarray scanner (G2505B; Agilent Technologies) and signal intensities were extracted and normalized within each array using Feature Extraction version 9.5 (Agilent Technologies). Gene intensities of <100 in replicates were neglected, for these are not significant. Signal intensities among biological replicates per infection were normalized according to the quantile method for standardization (global scaling) using EXPANDER6. Significance analysis was performed using the LIMMA function in Multiple experiment Viewer. For each infection experiment the fold change (FC) of each gene was determined by dividing its expression level in the inoculated plants by its expression level in mock-inoculated plants and taking its Log base 2. Differentially expressed genes (DEGs) were defined as genes with a false discovery rate (FDR) of <0.05 and a cut-off value of Log_2_FC <−1.5 or Log_2_FC >1.5. Loci above the FDR threshold or below the Log_2_FC cut-off were considered as not differentially expressed. The data have been deposited in NCBI’s Gene Expression Omnibus and are accessible through GEO Series accession number GSE133268. Gene Ontology (GO) enrichment analysis was performed using the Parametric Analysis of Gene Set Enrichment (PAGE) of agriGO (Kim and Volsky, 2005; Du et al., 2010). MapMan (Thimm et al., 2004) was used to visualize the expression of biotic stress related genes and genes involved in hormonal pathways in rice.

### RNA Extraction, cDNA Synthesis, and qRT-PCR

RNA extraction was performed with the RNeasy Plant Mini kit (Qiagen Hilden, Germany), followed by DNase treatment (DNaseI, Thermo Scientific) and cDNA synthesis using the Tetro cDNA Synthesis Kit (Bioline), as described by Kyndt et al. (2017). Two internal reference genes OsEXP and OsEXPnarsai were used to normalize the expression levels of rice genes (Nahar et al., 2012).

## Results

### Preliminary Hormone Measurements Show Minor JA Accumulation Upon *P. Arrhenomanes* Infection in Rice Roots

**Figure 1:**
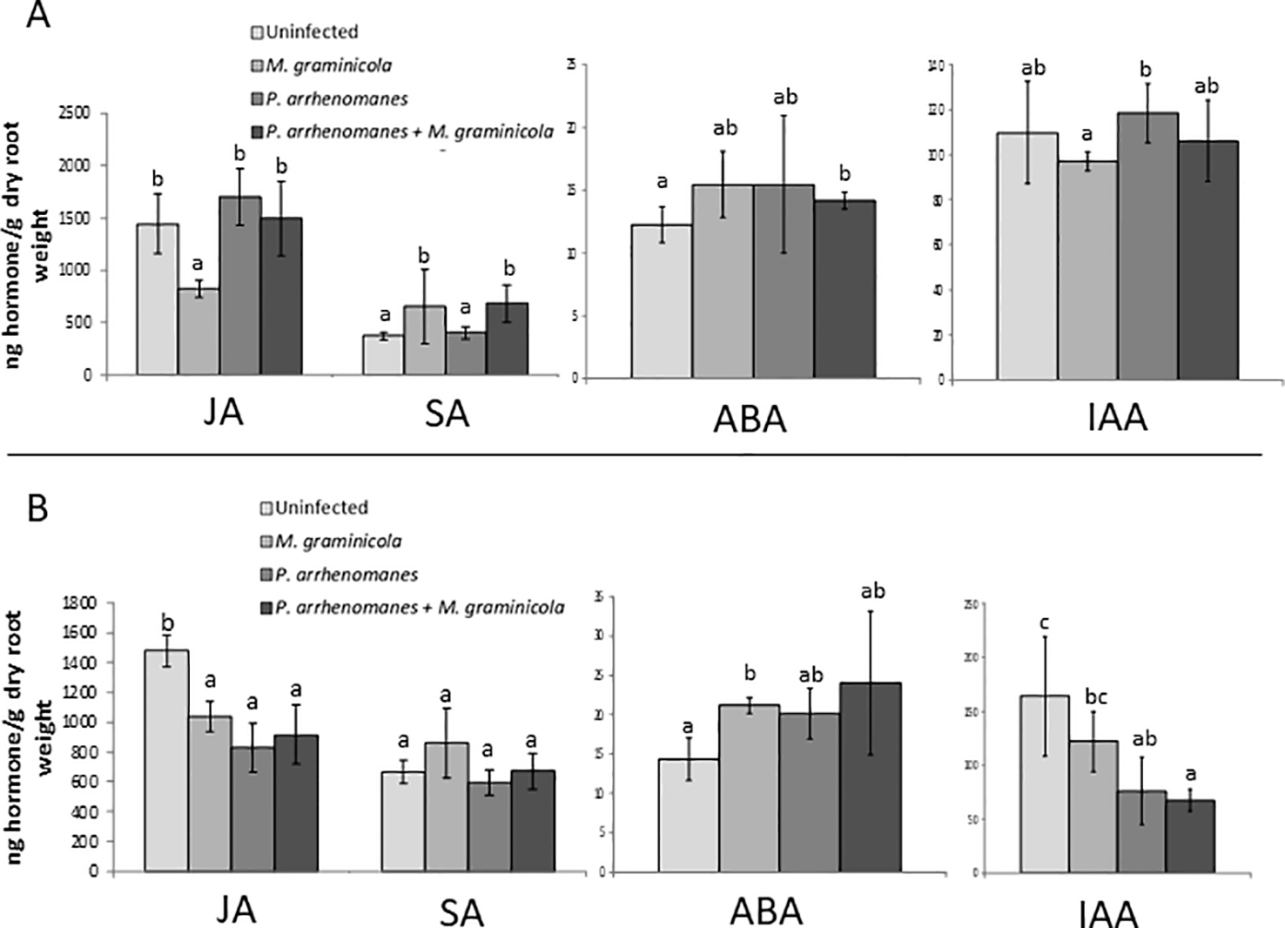
Hormone levels of jasmonic acid (JA), salicylic acid (SA), abscisic acid (ABA), and indole-3-acetic acid (IAA) in the roots of rice variety IR81413-BB-75-4 at 11 days **(A)** and 13 days **(B)** post germination (dpg). **(A)** corresponds with 4 days post oomycete inoculation (dpoi) and 3 days post nematode inoculation (dpni), **(B)** corresponds with 6 days post oomycete inoculation (dpoi) and 5 days post nematode inoculation (dpni). Treatments are: uninfected roots, single *M. graminicola* inoculation; single *P. arrhenomanes* inoculation; and *P. arrhenomanes* prior to *M. graminicola* inoculation. Bars represent mean ± standard deviation (SD) of three biological replicates, each consisting of a pool of six root systems. Different letters indicate statistical significant (α = 0.05) differences for that hormone at that time point.

Previously, we have reported that prior infection by the oomycete *P. arrhenomanes* consistently reduces the penetration rate and development of the root-knot nematode *M. graminicola* in rice roots, even under different infection and plant growth conditions (Verbeek et al., 2016). To elucidate a potential mechanism behind this antagonism, we decided to investigate the levels of a set of plant hormones, namely JA, SA, IAA—an important AUX—and ABA, upon single and double inoculations, using root samples taken at two time points (11 and 13 days post germination, dpg) during the greenhouse experiment described in detail in Verbeek et al. (2016). Results, shown in **Figure 1**, revealed JA levels in the roots are significantly reduced at 3 days post single nematode inoculation (11 dpg) compared to the un-inoculated control (**Figure 1A**). Four days after single *P. arrhenomanes* inoculation, a minor JA increase was observed. However, in double inoculated roots, JA levels did not differ from the un-inoculated control. At 13 dpg, JA levels were significantly lower in all single or double inoculated plants compared to the control (**Figure 1B**). The level of SA was significantly higher at 11 dpg in single nematode and double inoculated plants, compared to control and single *P. arrhenomanes* inoculated roots. Although not statistically significant, this trend was still visible at 13 dpg. ABA levels were very low in the rice roots, and only upon single nematode inoculation a significantly increased ABA level was observed at 13 dpg, confirming observations of Kyndt et al. (2017). Although fairly constant levels of IAA were detected at 11 dpg, at 13 dpg the IAA level dropped significantly in single *P. arrhenomanes* inoculated as well as in double-inoculated plants (**Figure 1B**).

Based on these data and the well-known importance of JA for basal defense against this nematode (Nahar et al., 2011), we hypothesized that an early JA accumulation—even earlier than the 4 days post oomycete inoculation time point analyzed in this greenhouse experiment—might be responsible for reduced nematode infection upon *P. arrhenomanes* infection in rice roots. This hypothesis was strengthened by the fact that the nematode is no longer able to suppress JA levels in double-inoculated plants (**Figure 1A**).

### The JA Pathway Is Activated Early Upon *P. Arrhenomanes* Inoculation in Rice, as Part of a Plant Defense Response

Based on the above-described observations we hypothesized that JA might be accumulating very early upon *P. arrhenomanes* inoculation. To test this hypothesis, hormone measurements were done under controlled *in vitro* conditions [as described in De Vleesschauwer et al. (2012)], as these are more adequate for standardized infection pressure, at both 2 and 4 dpi.

**Figure 2:**
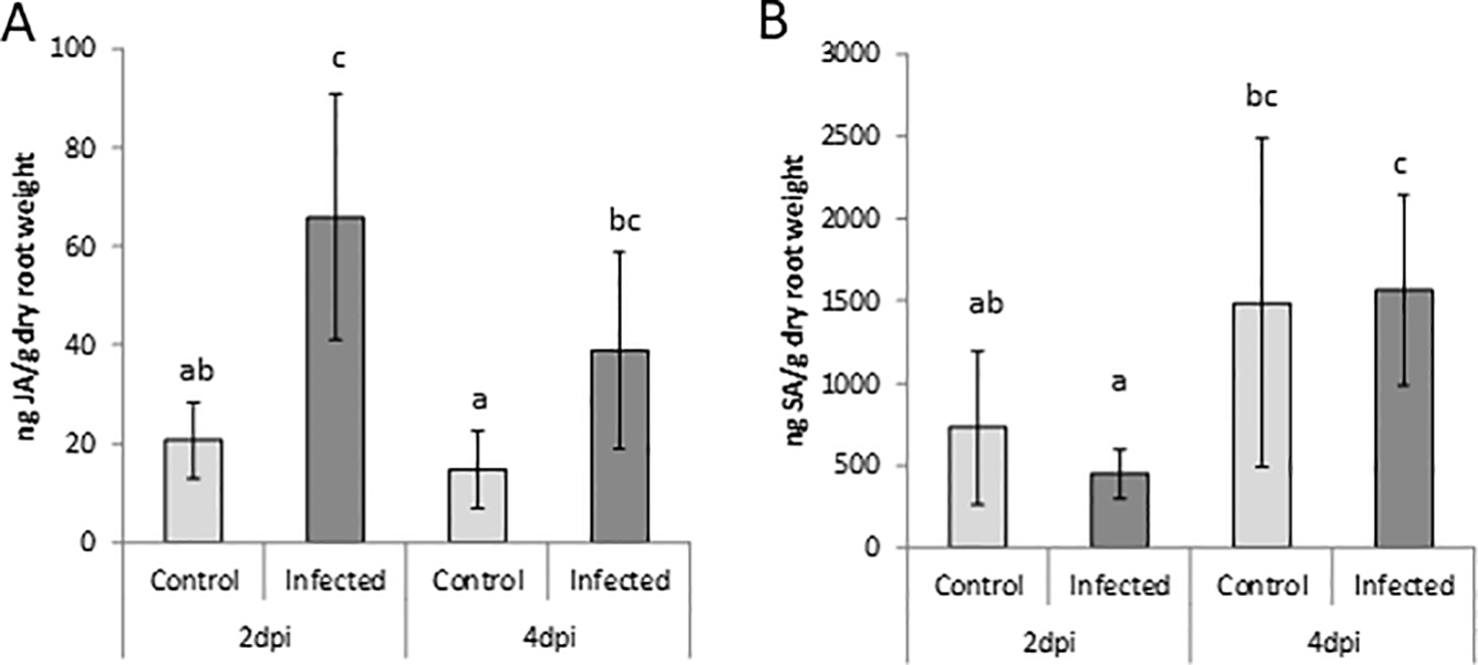
Jasmonic acid (JA; **A**) and salicylic acid (SA; **B**) levels in roots of Nipponbare plants grown on Gamborg B5 medium at 2 and 4 days post *P. arrhenomanes* inoculation compared to uninfected roots. Statistics were performed using the Mann-Whitney test (α = 0.05). Bars represent mean ± SD of five biological replicates, each consisting of a pool of 12 root systems. Different letters represent significant differences.

Probably due to the young tissue, only SA and JA levels were above the detection limit in this experiment. The data confirmed a strong accumulation of JA at 2 dpi with *P. arrhenomanes* and a lower but still significant accumulation at 4 dpi, whereas SA levels were unaffected by *P. arrhenomanes* infection in rice roots (**Figure 2**), as also observed in the greenhouse experiment (**Figure 1**).

**Figure 3:**
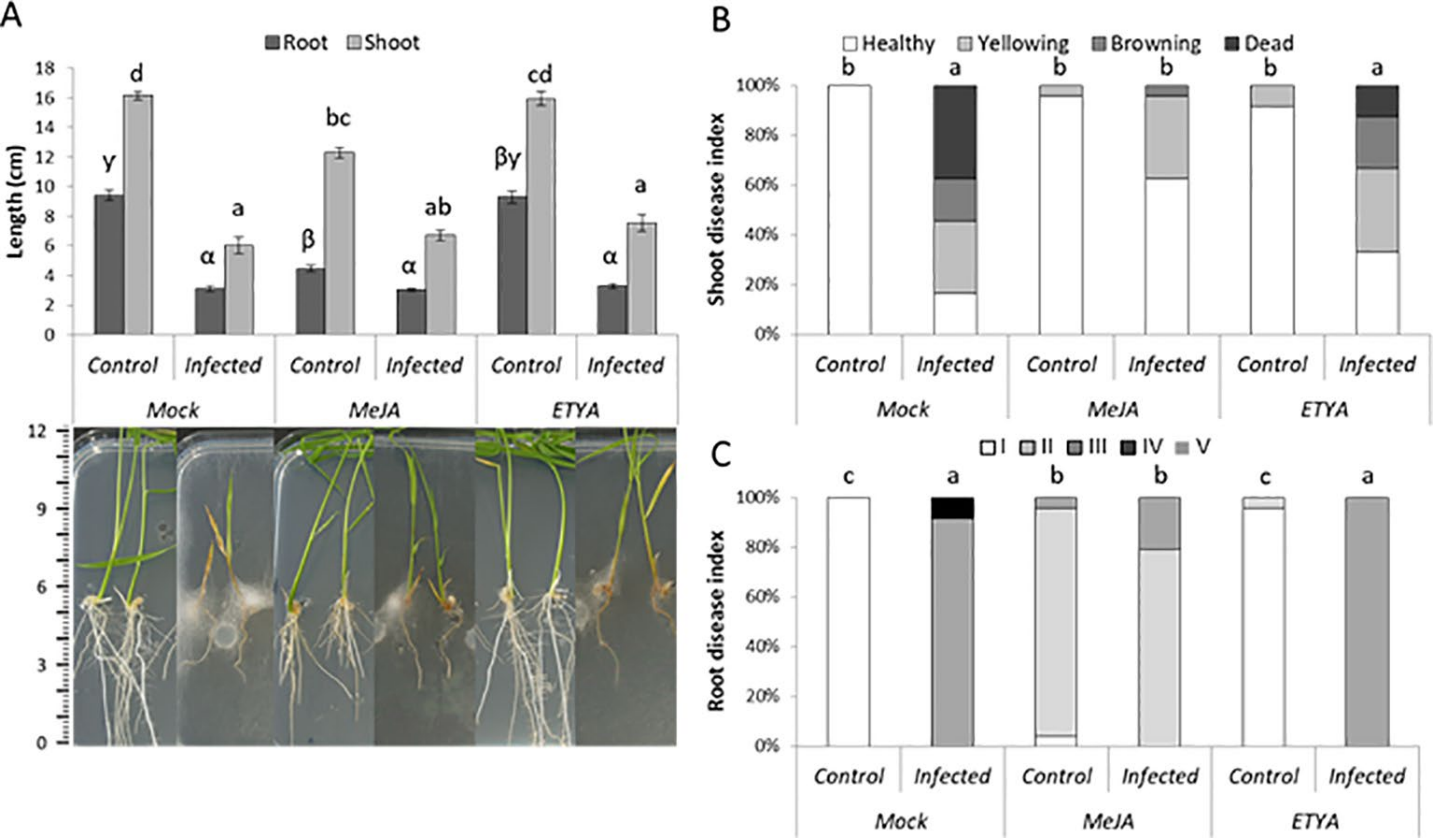
Effect of exogenous application of 10µM JA biosynthesis inhibitor ETYA and 25µM MeJA on *P. arrhenomanes*induced disease symptoms at 10 dpi, compared to non-treated rice seedlings. **(A)** root and shoot length with representative pictures of plants, **(B)** shoot disease index, and **(C)** root disease index. Root disease index: (I) root length (RL) > 60% of the respective mock treatment, necrosis covering <20% of total root area; (II) RL >60%, necrosis >20%; (III) RL 20% to 60%; (IV) RL <20%, necrosis <75%, and (V) RL <20%, necrosis >75%. Error bars represent the standard error (SE). An indicative scale bar (in cm) is shown on the left. Different letters represent statistically significant differences among the treatments at α = 0.05, P ≤ α performed by Mann–Whitney non-parametric tests. In **(A)**, statistical differences for shoot data are shown in Roman letters, while root differences are shown in Greek letters.

**Figure 4:**
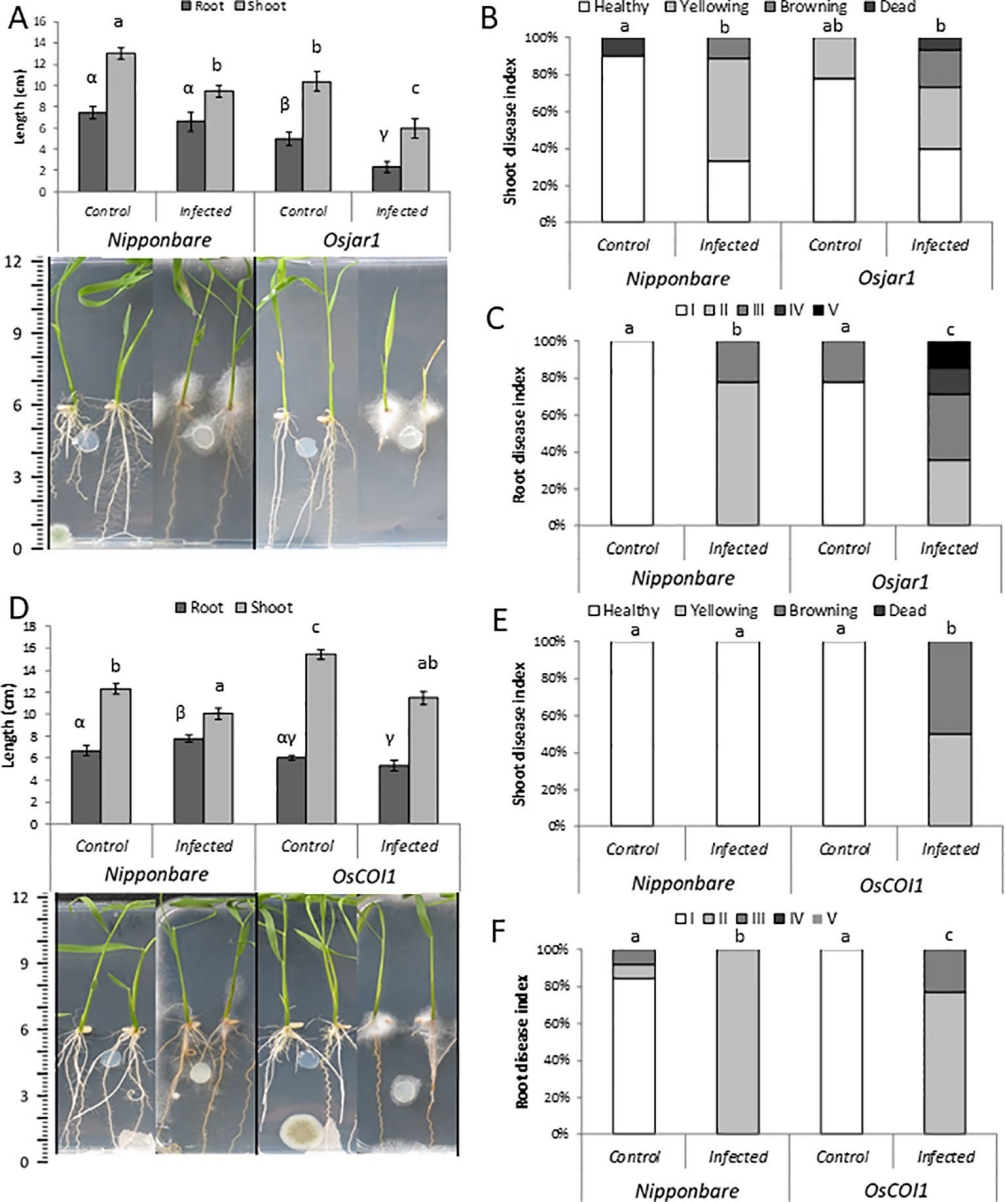
Infection of *P. arrhenomanes* on Nipponbare and *Osjar1* mutant **(A**–**C)**, and on the *OsCOI1* RNAi-line **(D**–**F)** at 7 days post *P. arrhenomanes* inoculation (n = 18). **(A**, **D)** root and shoot length with representative pictures of plants; **(B**, **E)** shoot disease index; **(C**, **F)** root disease index. Root disease index: (I); root length (RL) > 60% of the respective mock treatment, necrosis covering <20% of total root area, (II); RL >60%, necrosis >20%, (III); RL 20% to 60%, (IV); RL <20%, necrosis <75%, and (V); RL <20%, necrosis >75%. Different letters represent statistically significant differences among the treatments at α = 0.05, *P*≤ α performed by Mann-Whitney non-parametric tests. Error bars represent the SE. An indicative scale bar (in cm) is shown on the left. In **(A)**, statistical differences for shoot data are shown in Roman letters, while root differences are shown in Greek letters.

Next, we investigated the role of JA in basal plant defense against *P. arrhenomanes* by amending the growth medium with MeJA or ETYA (jasmonate biosynthesis inhibitor). In addition, the susceptibility of the JA-insensitive *Osjar1* mutant line and the *OsCOI1* RNAi-line was tested. Confirming the well-known inhibitory effect of JA on root growth, supply of MeJA significantly shortened rice root length with 47% (**Figure 3A**). Amending the plant growth medium with 25 µM MeJA significantly reduced the shoot and root disease index of the *P. arrhenomanes* inoculated plants. However, supply of ETYA did not significantly affect *Pythium*-induced disease symptoms in rice seedlings. The *Osjar1*-mutant, which contains lower levels of JA-Ile and is less sensitive to JA (Riemann et al., 2008; Fukumoto et al., 2013), was slightly more susceptible to *P. arrhenomanes* than wild-type plants (**Figure 4**). Similarly, the JA-insensitive *OsCOI1* RNAi-line (Yang et al., 2012), was significantly more susceptible to *P. arrhenomanes* than the control line (**Figure 4**).

These data demonstrate that the JA pathway is induced upon *P. arrhenomanes* infection in rice roots as part of a plant defense response and lead to the hypothesis that JA induction would be the causal mechanism behind the antagonistic interaction with *M. graminicola*, reported in Verbeek et al. (2016).

### The Importance of the JA Pathway in the Antagonism Between *P. arrhenomanes* and *M. graminicola* in Rice Roots

**Figure 5:**
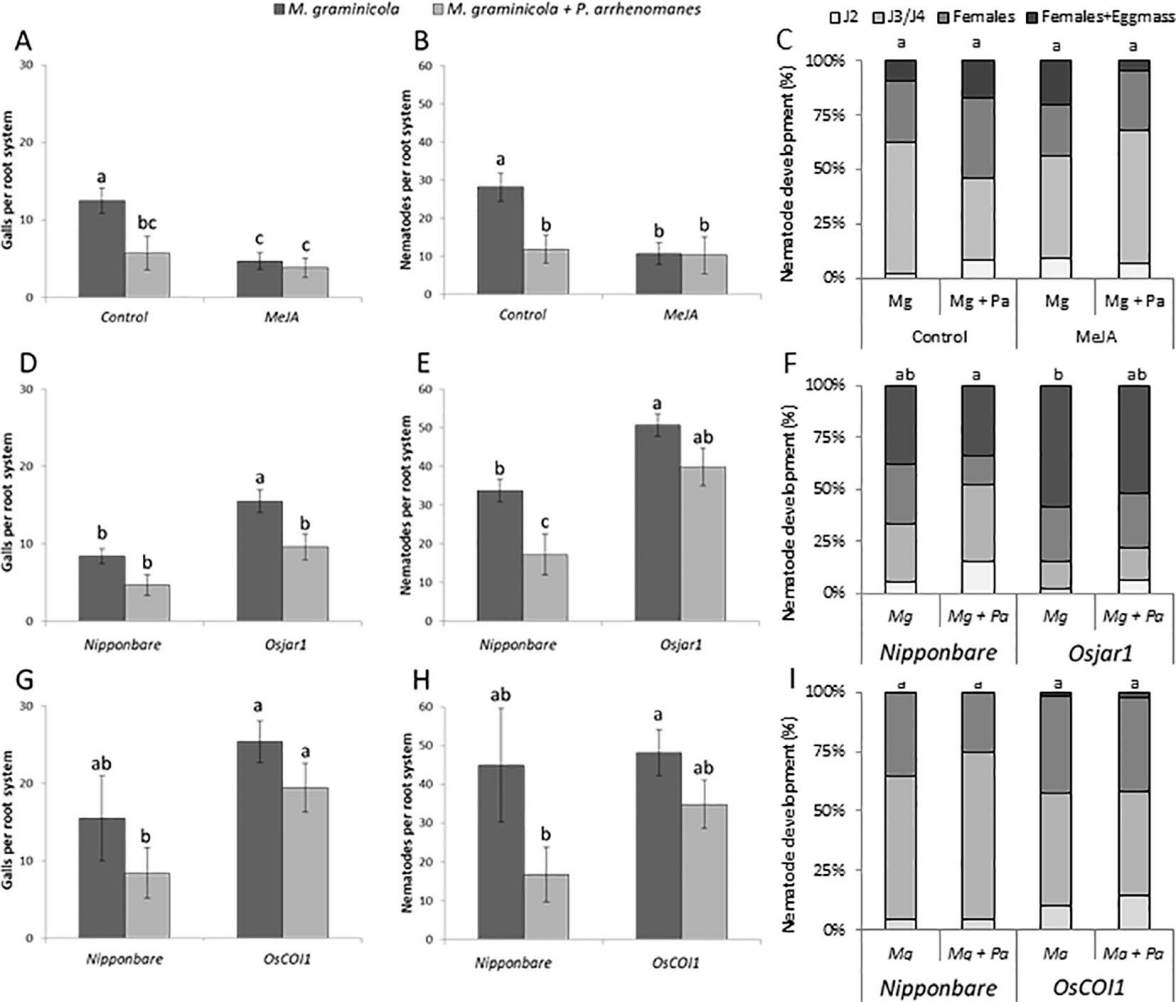
Gall formation **(A**,**D**,**G)**, nematode establishment **(B**, **E**, **H)**, and nematode development **(C**, **F**, **I**) in wild-type (Nipponbare); after treatment with MeJA (**A**–**C**; n = 8), in the JA biosynthesis mutant *Osjar1* (NC2728; **(D**–**F)**; n = 8), and in the JA signaling *OsCOI1* RNAi-line (**G**–**I**; n = 8). Statistics were performed using the Mann-Whitney test (α = 0.05). Different letters represent significant differences. Error bars represent the SE. Statistics for **(C**, **F**, **I)** were performed by giving each group a total score; where the percentage of each stage has a value; juvenile stage 2 (J2) = 1; juvenile stages 3 and 4 (J3/J4) = 2; females = 3; and females with eggmass = 4.

A set of double inoculation experiments was done with both *P. arrhenomanes* and *M. graminicola*. In these experiments, the role of JA in the antagonistic interaction between *P. arrhenomanes* and *M. graminicola* was evaluated by working with the previously described JA-mutants or exogenous supply of MeJA. An inoculation substrate optimized for *M. graminicola* infection, designed by Reversat et al. (1999), was used (see Methods and Timeline in **Figure S1**). Confirming the importance of JA in rice defense against this nematode (Nahar et al., 2011), the *Osjar1* mutant was more susceptible to *M. graminicola* (**Figures 5D–I**), whereas foliar application with MeJA significantly reduced the level of *M. graminicola* infection. However, the *OsCOI1* RNAi line was only marginally more susceptible to nematodes than wild-type plants.

Although not always statistically significant, a reduced number of galls and number of penetrating nematodes was seen in the double-inoculated plants in comparison with single-nematode inoculated plants. Contrary to our previous observations in greenhouse experiments (Verbeek et al., 2016), no significant inhibitory effect of *Pythium* infection on nematode development was observed in the here-used substrate (**Figure 5**).

The reduction in nematode penetration and gall formation, triggered by MeJA application, phenocopies the reduction caused by *P. arrhenomanes* infection (**Figures 5A, B**). Furthermore, *P. arrhenomanes* infection on MeJA treated plants does not further reduce gall formation by *M. graminicola* (**Figures 5A, B**). Noteworthy, MeJA treatment reduces root growth, but when *M. graminicola* infection was expressed as nematodes per cm root, the same trends were seen (**Figure S2**). When considering the number of galls, in the *Osjar1* mutant and the *OsCOI1* RNAi-line, the antagonism was less strong compared to wild-type plants (**Figures 5D–I**). Taken together, these data indicate that the antagonism between these two pathogens in rice roots is partially dependent on JA signaling.

To further explore which JA-dependent or JA-independent defense mechanisms could be responsible for the antagonism, micro-array analysis was used to investigate gene expression in rice roots at 2 days after *P. arrhenomanes* inoculation. The gene expression profile revealed that 5147 rice genes are DEGs in inoculated roots compared to the mock inoculated control (FDR <0.05). Genes belonging to GO-categories “signaling,” “response to stimulus,” and “catalytic activity” were most upregulated, while genes associated with “structural molecule activity,” “organelle,” “macromolecular complex,” and “cellular component organization” were most downregulated (**Figure S3**). Genes involved in AUX biosynthesis and signaling were generally downregulated upon *Pythium* infection in rice roots, a phenomenon which was already detected in the hormone measurements reported in **Figure 1**(**Table S1**). Confirming our hypothesis of an early JA activation, transcription of JA biosynthesis and signaling genes—e.g. genes encoding JAZ—was strongly upregulated at 2 dpi with *P. arrhenomanes* (**Table S2**).

Among the strongest down-regulated “biotic stress-related” genes were many dirigent genes, two nucleotide-binding site leucine-rich repeat (LRR) disease resistance genes, and two LRR protein genes.

**Figure 6:**
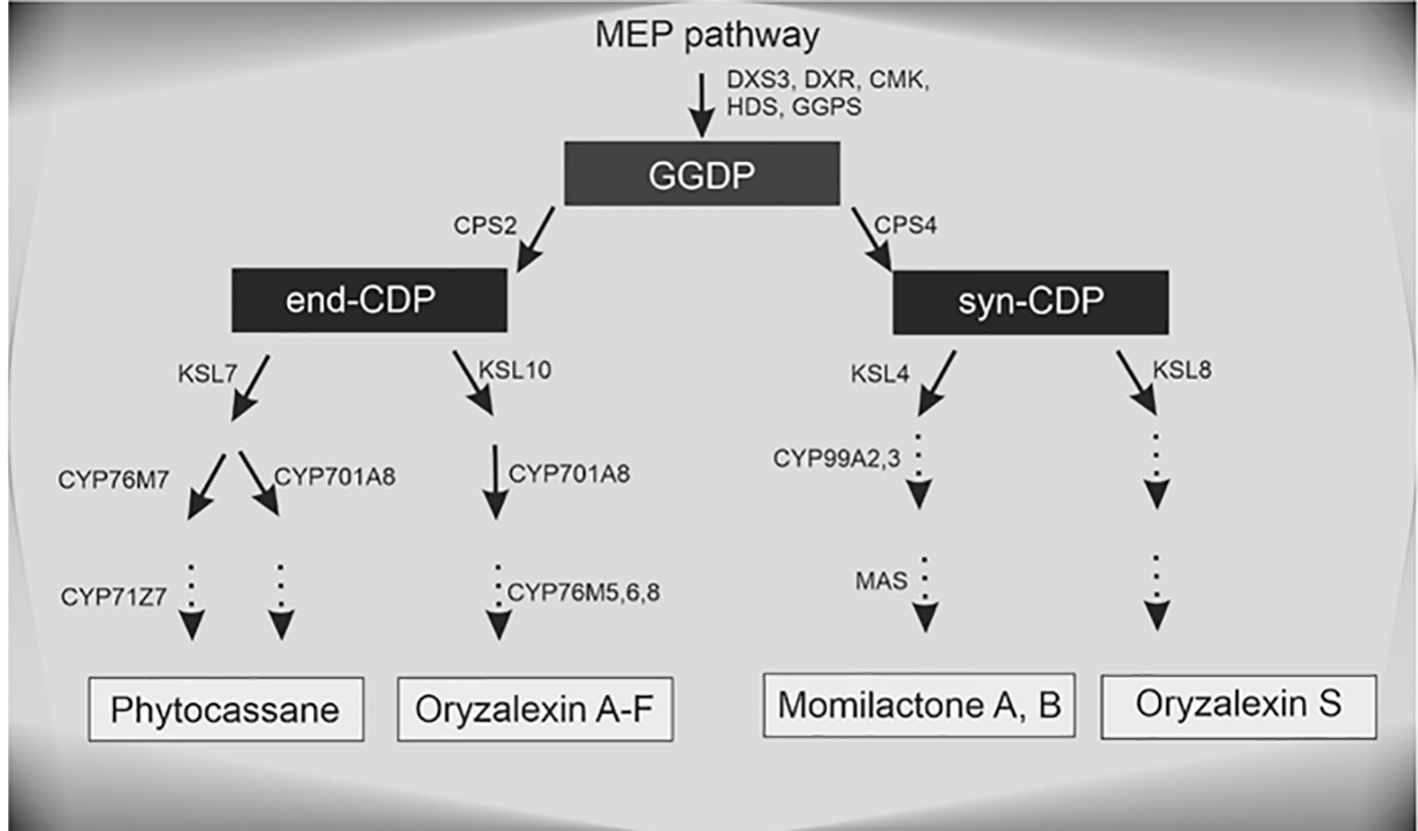
Methylerythritol 4-phosphate (MEP) pathway and subsequent diterpenoid phytoalexin biosynthesis in rice, based on Miyamoto et al. (2014).

The strongest upregulated biotic stress genes all appear to be involved in the biosynthesis of diterpenoid phytoalexins (DP) such as phytocassanes, oryzalexins, and momilactones *via* the methylerythritol phosphate (MEP) pathway (**Figure 6**, **Table 1**). **Table 2** lists TFs that were reported to be involved in the regulation of DP production. OsTGAP1 is a chitin-elicitor induced basic leucine zipper (bZIP) TF involved in the regulation of momilactone and phytocassane biosynthesis in rice cells (Okada et al., 2009). OsbZIP79 is a protein that interacts with OsTGAP1 and acts as a suppressor of chitin-elicitor inducible expression of DP (Miyamoto et al., 2014). These regulators do not seem to be involved in the *Pythium*-induced DP induction. It is clear that especially the DP factor (DPF) is strongly upregulated by *P. arrhenomanes* inoculation. Strikingly, almost all MEP and DP biosynthesis genes listed in **Table 1** are co-expressed with DPF. Interestingly, also other genes co-expressed with DPF as reported in Yamamura et al. (2015) were upregulated by *P. arrhenomanes* inoculation (see DPF regulon in **Table 2**). DPF is known to specifically accumulate in roots and husks and is induced by MeJA. Recently it was shown by Fukushima et al. (2016) and Akagi et al. (2014) that the WRKY45-WRKY62 heterodimer acts as a strong activator of DP biosynthetic genes. The simultaneous introduction of WRKY45 and WRKY62 resulted in a strong upregulation of the DPF promoter. These TFs were previously described to be induced by benzathiadiazole, an analogue of SA (Fukushima et al., 2016). Our data reveals that both TFs are induced in similar levels by *P. arrhenomanes* infection. Also activation of the OsMKK4-OsMPK3/OsMPK6 cascade leads to DP accumulation (Kishi-Kaboshi et al., 2010). Expression of OsWRKY53, one of the components of the downstream signaling of this cascade (Chujo et al., 2014), was also induced by *P. arrhenomanes* infection (**Table 2**). Next to the DPF regulon, also the recently described TF OsERF83 is strongly upregulated by *P. arrhenomanes* infection. This TF is transiently induced by treating rice seedlings with SA or ethephon and more constantly triggered by MeJA (Tezuka et al., 2019). PR genes upregulated in rice lines overexpressing OsERF83 are also transcriptionally activated upon *P. arrhenomanes* infection (**Table 2**).

**Table 1:** Overview of transcriptional changes (log2 fold-change) as observed by micro-array analysis of *Pythium arrhenomanes* infected rice roots, at 2 days post inoculation in comparison with mock-inoculated roots.

Gene symbol	Description	RAP ID	MSU	Log2 fold-change	Co-expressed with DPF
*MEP pathway genes*					
OsDXS3	DXP synthase	Os07g0190000	LOC_Os07g09190	9.28	x
OxDXR	DXP reductoisomerase	Os01g0106900	LOC_Os01g01710	4.91	x
OsCMK	CDP-ME kinase	Os01g0802100	LOC_Os01g58790	3.73	
OsHDS	HMBDP synthase	Os02g0603800	LOC_Os02g39160	4.52	
OsGGPS	GGDP synthase	Os07g0580900	LOC_Os07g39270	4.53	x
*Diterpenoid phytoalexins*					
OsCPS2	ENT-COPALYL DIPHOSPHATE SYNTHASE 2	Os02g0571100	LOC_Os02g36210	9.67	x
OsCPS4	ENT-COPALYL DIPHOSPHATE SYNTHASE 4	Os04g0178300	LOC_Os04g09900	7.17	x
OsKSL4	ENT-KAURENE SYNTHASE 4	Os04g0179700	LOC_Os04g10060	7.86	x
OSKSL7/OsDTC1	ENT-KAURENE SYNTHASE 7	Os02g0570400	LOC_Os02g36140	7.81	x
OSKSL8/OsDTC2	ENT-KAURENE SYNTHASE 8	Os11g0474800	LOC_Os11g28530	5.04	x
OsKSL10	KAURENE SYNTHASE-LIKE 10	Os12g0491800	LOC_Os12g30824	9.43	x
CYP71Z2	P-450 71Z2	Os07g0217600	LOC_Os07g11739	10.01	x
CYP71Z7	P-450 71Z7	Os02g0570700	LOC_Os02g36190	9.08	x
CYP76M5	P-450 76M5	Os02g0569000	LOC_Os02g36030	9.57	x
CYP76M6	P-450 76M6	Os02g0571900	LOC_Os02g36280	7.46	x
CYP76M7	P-450 76M7	Os02g0569900	LOC_Os02g36110	8.66	x
CYP76M8	P-450 76M8	Os02g0569400	LOC_Os02g36070	6.08	x
OsKOL4/CYP701A8	ENT-KAURENE OXIDASE 4	Os06g0569500	LOC_Os06g37300	8.95	x
CYP99A2	P-450 99A2	Os04g0180400	LOC_Os04g10160	9.00	x
CYP99A3	P-450 99A3	Os04g0178400	LOC_Os04g09920	8.03	x
OsMAS	MOMILACTONE A SYNTHASE	Os04g0179200	LOC_Os04g10010	6.35	x

Genes related to the methylerythritol 4-phosphate (MEP) pathway and subsequent diterpenoid phytoalexin biosynthesis in rice are shown. x indicates that these genes are known to be co-expressed with DPF. RAP, Rice Annotation Project-locus number; MSU, MSU locus number; DPF, diterpenoid phytoalexin factor.

**Table 2:** Overview of transcriptional changes (log2 fold-change) in the expression of transcription factors involved in diterpenoid phytoalexin biosynthesis and genes known to be regulated by DPF and OsERF83, as observed by micro-array analysis of Pythium arrhenomanes infected rice roots at 2 days post inoculation, in comparison with mock-inoculated roots.

Gene symbol	Description	RAP ID	MSU	Log2 fold-change	References
*Transcription factors implicated in phytoalexin production*					
BZIP37/OsTGAP1	b-ZIP TRANSCRIPTION FACTOR 37	Os04g0637000	LOC_Os04g54474	0.33	Okada et al., 2009; Miyamoto et al., 2014
DPF	DITERPENOID PHYTOALEXIN FACTOR	Os01g0196300	LOC_Os01g09990	6.02	Yamamura et al., 2015
OsbZIP79	b-ZIP TRANSCRIPTION FACTOR 79	Os11g0152700	LOC_Os11g05480	-0.27	Miyamoto et al., 2014
OsWRKY45	WRKY GENE 45	Os05g0322900	LOC_Os05g25770	2.36	Akagi et al., 2014
OsWRKY62	WRKY GENE 62	Os09g0417800	LOC_Os09g25070	2.60	Fukushima et al., 2016
OsWRKY53	WRKY GENE 53	Os05g0343400	LOC_Os05g27730	2.15	Chujo et al., 2014
*DPF regulon*					Yamamura et al., 2015
DPF	DITERPENOID PHYTOALEXIN FACTOR	Os01g0196300	LOC_Os01g09990	6.02	
RSOsPR10	ROOT SPECIFIC PR10	Os12g0555000	LOC_Os12g36830	6.26	
OsPR10b	PATHOGENESIS-RELATED GENE 10B	Os12g0555200	LOC_Os12g36850	6.12	
FAD2-like2	FATTY ACID DESATURASE	Os07g0416900	LOC_Os07g23410	5.25	
OsPDR5	PLEIOTROPIC DRUG RESISTANCE 5/putative ABC transporter	Os07g0522500	LOC_Os07g33780	3.44	
*OsERF83 regulon*					Tezuka et al., 2019
OsERF83	ETHYLENE RESPONSE FACTOR 83	Os03g0860100	LOC_Os03g64260	5.90	
OsPR1#74/PR1A	PATHOGENESIS-RELATED GENE 1A	Os07g0129200	LOC_Os07g03710	2.39	
PR2/GNS6	BETA 1-3 GLUCANASE6	Os01g0940800	LOC_Os01g71350	4.81	
PR3/CHT5	CHITINASE 5	Os04g0494100	LOC_Os04g41680	8.05	
PR5/thaumatin/TLP	THAUMATIN-LIKE	Os03g0663500	LOC_Os03g46070	3.79	
OsPR10b	PATHOGENESIS-RELATED GENE 10B	Os12g0555200	LOC_Os12g36850	6.12	
RIR1b	DEFENSE RELATED GENE RIR1b	Os10g0569400	LOC_Os10g41980	2.84	

These data were validated and extended by qRT-PCR targeting jasmonate biosynthesis gene allene oxide synthase 2 (*OsAOS2*) and pathogenesis-related gene 10 (*OsPR10*), in single and double-inoculated rice plants. At this early time point after nematode inoculation (1 day), both genes were activated in the rice roots, probably corresponding to an early plant defense response triggered by nematode migration. Our data confirmed strongly induced expression of both genes at two days after *P. arrhenomanes* infection (**Figure S4**).

## Discussion

In our previous work we have documented the antagonistic interaction between *P. arrhenomanes* and *M. graminicola* in rice grown under different experimental conditions (Verbeek et al., 2016). We observed that prior inoculation by *P. arrhenomanes* results in a reduction of the number of established nematodes, gall formation, and slower nematode development in rice roots. Samples taken during the previously described greenhouse experiment (Verbeek et al., 2016) were in this manuscript analyzed further by executing phytohormone measurements and gene expression analyses in rice roots.

### Confirming the Dynamic Role of JA in the Warfare Between Rice and *M. graminicola*

Our data showed that JA levels are suppressed in rice roots upon *M. graminicola* infection. Ji et al. (2013) already reported that transcription of JA biosynthesis genes is locally suppressed in nematode-induced giant cells. Nahar et al. (2011) observed a similar phenomenon in very young gall tissue (1–2 dpi) and provided data showing that the JA pathway plays a pivotal role in rice defense against *M. graminicola*, information which was confirmed and extended here by the increased susceptibility of the JA-insensitive *Osjar1* mutant (**Figure 5**). Nevertheless, at 3 dpi, JA has also been reported to be accumulating in gall tissue (Yimer et al., 2018). Transcriptome data of Kyndt et al. (2012) showed that while some JA related genes are suppressed, others are activated in 3 dpi galls. Our data show a similar view where *OsAOS2* was also upregulated at 1 day after nematode infection (**Figure S4**). This activation is most probably due to minor root damage upon nematode penetration and gall expansion. The JA level in nematode-infected roots hence strongly varies depending on the tissue and time point that is being evaluated.

### *Pythium* Infection Activates JA and Related Plant Defense Responses in Roots, Which Could Hamper Subsequent *M. graminicola* Infection

Our results showed a slight JA accumulation upon *P. arrhenomanes* infection at 4 days after inoculation. However, more importantly, JA suppression by *M. graminicola* infection was no longer observed when the roots were prior infected with *P. arrhenomanes* (**Figure 1**). Based on this observation, we hypothesized that JA might play a role in the antagonism, and a set of experiments was executed to confirm the induction of the JA pathway in host roots upon *P. arrhenomanes* infection. Two days after *P. arrhenomanes* inoculation on *in vitro* grown rice seedlings a clear induction of JA biosynthesis and JA signaling genes was detected using micro-array analysis, an observation which was correlated with increased JA levels in the roots (**Figure 2**). Increases in JA were previously described upon *Pythium* infection in moss (Oliver et al., 2009) and apple (Shin et al., 2014). The JA peak in rice roots appeared to be transient, as the boosted endogenous JA peak vanished at later time points (**Figures 1**and **2**). A similar transient peak was observed by Shin et al. (2014), who observed that transcription of an apple JA biosynthesis *AOS* gene peaked within 48 h after inoculation with *P. ultimum* and receded within 96 h. Interestingly, upon double inoculation of *P. arrhenomanes* and *M. graminicola* the JA levels in the roots can no longer be suppressed by the nematode (**Figure 1**).

### The Role of JA in Plant Defense Against *P. arrhenomanes*

Confirming observations from the interaction between *P. irregulare* and *Arabidopsis* (Staswick et al., 1998), the JA pathway was found to negatively affect oomycete infection in rice roots. MeJA-amended rice seedlings were less susceptible to *P. arrhenomanes*, whereas increased susceptibility was observed in the *Osjar1* transgenic line and the *OsCOI1* RNAi-line (**Figure 4**). Noteworthy, application of a JA-biosynthesis inhibitor did not affect rice susceptibility to this oomycete, indicating that JA-signaling is more important for plant defense against this oomycete than actual JA levels. Interestingly, genes encoding proteins belonging to the JAZ family are strongly upregulated after *P. arrhenomanes* infection (**Table S2**). JAZ domains repress the transcription of JA responsive genes by binding to MYC2 domains. The SCF^COI1^E3 ligase complex, which perceives JA-Ile presence, binds to the Jas domain of JAZ proteins and marks them for proteasomal degradation (Pauwels and Goossens, 2011). An effector protein of the ectomycorrhizal fungus *Laccaria bicolor*, MiSSP7, interacts with PtJAZ6 (*Populus trichocarpa*) in order to prevent JA-induced degradation of PtJAZ6 (Plett et al., 2014). *P. arrhenomanes* might possess similar effectors that can stabilize the JAZ proteins, as a mechanism to prevent JA-related defense gene expression in the host.

In double-inoculated plants amended with MeJA, nematode infection is suppressed and *P. arrhenomanes* is not able to further increase defense (**Figure 5**), indicating that the JA pathway plays a role in the interaction. However, it could be argued that exogenous application of MeJA has prevented *P. arrhenomanes* colonization and in this way indirectly precluded any antagonistic effect on the nematode. Nevertheless, in this double-inoculation experiment, *P. arrhenomanes* was inoculated 3 days prior to MeJA application, and Van Buyten and Höfte (2013) showed that *P. arrhenomanes* colonizes the inner root tissues 2 days upon inoculation, which renders this hypothesis questionable. It is therefore most likely that *M. graminicola* is affected by the *P. arrhenomanes*-induced JA-defense mechanisms, up to a level that cannot be suppressed efficiently by the nematode. However, the fact that the antagonism is reduced but still observable upon genetic or chemical inhibition of this pathway reveals that additional JA-independent mechanisms seem to be involved.

### Transcriptional Changes Upon *Pythium* Infection in Rice: JA-Regulated Defense Responses, Involving DPF and ERF83

**Figure 7:**
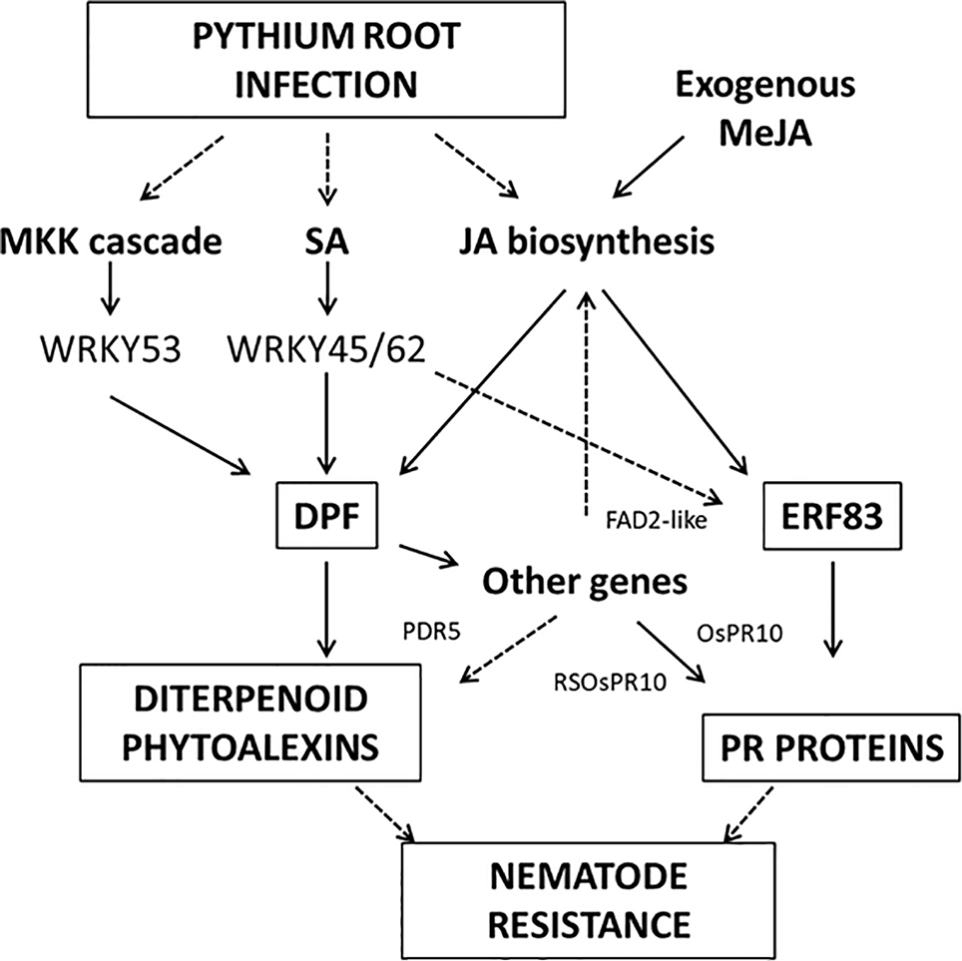
Speculative model showing how *P. arrhenomanes* infection can lead to the activation of genes involved in the biosynthesis of diterpenoid phytoalexins (DP) and PR proteins, resulting in induced nematode resistance.

Interestingly, the micro-array analysis of *P. arrhenomanes*-infected root systems revealed a significant induction of genes involved in DP biosynthesis. Phytoalexins are known to be synthesized in infected plants in response to infection and can be secreted from root tissue, for example to act as allelochemicals (Toyomasu et al., 2008). In literature, a clear correlation between resistance against nematodes and levels of different phytoalexins has been reported in different host plants, e.g. resistant lima bean (*Phaseolus lunatus*) infected by *Pratylenchus penetrans* produces the phytoalexin coumestrol and banana cultivars resistant to *Radopholous similis* accumulate significant amounts of phenalenone-type phytoalexins, which are derived from the phenylpropanoid pathway (Hölscher et al., 2014). Similarly, production and exudation of phytoalexins by soybean root tissues infected with the cyst nematode *Heterodera glycines* are also restricted to resistant cultivars (Huang and Barker, 1991). Overexpression of the *Arabidopsis* phytoalexin-deficient 4 gene (AtPAD4) enhances resistance in soybean roots in response to the phytoparasitic nematode species *Meloidogyne incognita* (root-knot nematode) and *H. glycines* (Youssef et al., 2013). Upon overexpression of the rice momilactone A synthase gene in soybean, the *H. glycines* female index decreased to 41% of the control (Matthews et al., 2013). Nevertheless, except for the observation that systemic tissues of *Hirschmaniella oryzae*-infected plants show enhanced expression of a terpene synthase (LOC_Os12g30824; OsKSL10), necessary for the biosynthesis of ent-sandaracopimaradiene, a precursor of the DPs oryzalexin A–F (Kyndt et al., 2013), nothing more is known about the role of rice phytoalexins in the interaction with plant-parasitic nematodes. The phenolic sakuranetin possesses strong antimicrobial activity against the blast fungus (Kodama et al., 1992), while DP compounds from rice exhibit antibiotic activity against *Rhizoctonia solani* (Koga et al., 1997). However, we found no evidence of induction of flavonoid phytoalexins by *P. arrhenomanes* infection. *NARINGENIN 7-O-METHYLTRANSFERASE (OsNOMT)*(Os12g0240900), the key gene in sakuranetin biosynthesis, was not upregulated. Most rice phytoalexins are diterpenoid compounds, including momilactones, phytocassanes, and oryzalexins, and all of them have been found in extracts of, and exudates from, the roots of rice seedlings (Toyomasu et al., 2008). Data on potential nematicidal or nematode-repelling effects of these compounds have, however, not been reported in scientific literature and deserve to be further explored. The DP pathway is known to be induced by OsTGAP1, the OsMKK4-OsMPK3/MPK6 pathway probably *via* OsWRKY53 activation, by MeJA reviewed by Miyamoto et al. (2014) and by the BTH-inducible WRKY45-WRKY62 heterodimer (Fukushima et al., 2016). The most important regulator of the diterpenoid pathway is the DPF (Yamamura et al., 2015). This TF is highly upregulated by *P. arrhenomanes* infection and is inducible by MeJA. In addition, WRKY45-WRKY62 heterodimer-induced and OsMKK4-induced phytoalexin production appears to be mediated by DPF (Yamamura et al., 2015; Fukushima et al., 2016). This may explain why *Osjar1-2* mutants inoculated with *Magnaporthe oryzae* could still produce DPs, showing that JA is not essential for DP biosynthesis (Shimizu et al., 2013). In this context it is interesting to notice that *P. arrhenomanes* could still reduce the numbers of nematode galls per root system in the *Osjar1* mutant. Also other DPF-regulated genes are highly induced upon *Pythium* infection. *Co1* or *FAD2-like1*, which is mainly expressed in roots, was also strongly induced by *P. arrhenomanes*. According to Yamamura et al. (2015) the protein encoded by *FAD2-like1* is homologous to OsFAD2, a fatty acid desaturase that is a key enzyme for the conversion of oleic acid (18:1) into linoleic acid (18:2). The protein may be involved in pathogen-induced JA biosynthesis through production of the precursor, linoleic acid. *OsPDR5* encodes a putative ABC transporter and may be involved in exporting antimicrobial metabolites, such as DPs (Yamamura et al., 2015). PR10b and RSOsPR10 are members of the PR10 family. RS0sPR10 is a root-specific protein that is specifically induced in roots in response to biotic and abiotic stress such as wounding (Bertini et al., 2019). Genes of several classes of PR proteins are also induced by OsERF83, a recently described TF with high similarity to the *Arabidopsis* TFs ERF15, ORA59, and ERF1 (Tezuka et al., 2019). OsERF83 is strongly expressed in roots and inducible by JA, SA, and ET. This TF and its regulon are upregulated by *Pythium* infection. A speculative model how DP biosynthestic genes and genes encoding PR proteins are upregulated upon *P. arrhenomanes* infection is presented in **Figure 7**.

### A Potential Role for Auxin in the Antagonism Between *P. arrhenomanes* and *M. graminicola* in Rice?

Next to the induction of JA, hormone analyses also showed that the IAA level in the root is depleted in *P. arrhenomanes* infected roots at 6 days after inoculation (13 dpg, **Figure 1**). Auxin is important for gall formation by *M. incognita* in *Arabidopsis*, and AUX transporters re-distribute AUX from the rest of the root system toward the gall (Grunewald et al., 2009; Kyndt et al., 2016). Also in rice, blocking AUX transport significantly reduces the *M. graminicola*-induced gall formation and delays nematode development (Yimer et al., 2018). Although further confirmation is needed, a depleted level of IAA in the plant caused by *P. arrhenomanes* infection could be the reason for the hampered nematode feeding site formation and nematode development as previously seen in double-inoculated plants under high *Pythium* infection pressure (Verbeek et al., 2016). In this previous publication, the antagonism of *P. arrhenomanes* against *M. graminicola* has been observed in a variety of different experimental set-ups, and appeared to be positively correlated with *P. arrhenomanes* infection pressure (Verbeek et al., 2016). In the current manuscript we confirmed a similar antagonism under controlled laboratory conditions. However, while ideal for nematode inoculations, the SAP-substrate seemed less suited for *P. arrhenomanes* infection and therefore resulted in a weaker antagonism against *M. graminicola*, as compared to the previously published data. For example, under greenhouse conditions the antagonism was still observed when *M. graminicola* was inoculated six days after *P. arrhenomanes* inoculation (Verbeek et al., 2016), while in the SAP substrate the antagonism was only observable when using a maximum four-day interval between both inoculations (data not shown). Additionally, negative effects were only consistently observed on gall formation and nematode penetration, while nematode development was in some experiments unaffected under laboratory conditions (**Figure 5**). As observed before (Verbeek et al., 2016), the strength of the antagonism versus nematode infection seems strongly dependent on the infection pressure of *P. arrhenomanes*.

## General Conclusion

Altogether, the here-reported data indicate that an early activation of the host jasmonate pathway upon *P. arrhenomanes* infection, correlated with the activation of ERF83 and DP genes, is at least partially responsible for a reduced number of nematodes infecting the root. Although further experimental confirmation is needed, an additional effect on nematode feeding site formation could potentially be related to AUX depletion upon *P. arrhenomanes* infection in the roots.

## Data Availability Statement

The datasets generated for this study can be found in the GEO: number GSE133268.

## Author Contributions

RV, GG, MH, and TK designed the study. EB and DV developed and performed the *in vitro Pythium* experiments. JB, DV, and SK performed the microarray study. AH and KD performed the hormone measurements. RV and MA performed the co-inoculation experiments. RV performed the statistical analyses and wrote the paper. GG, MH, and TK helped in revising the paper. All authors read and approved the final manuscript.

## Funding

This research was supported by FWO-project G.0315.13 N and GOA-project G.03013.

## Conflict of Interest

The authors declare that the research was conducted in the absence of any commercial or financial relationships that could be construed as a potential conflict of interest.
