# Supplementary figures and images for: Jasmonate-Induced Defense Mechanisms in the Belowground Antagonistic Interaction Between Pythium arrhenomanes and Meloidogyne graminicola in Rice
Source: Front Plant Sci. 2019 Nov 22;10:1515. doi: 10.3389/fpls.2019.01515 (PMC6883413; doi:10.3389/fpls.2019.01515)

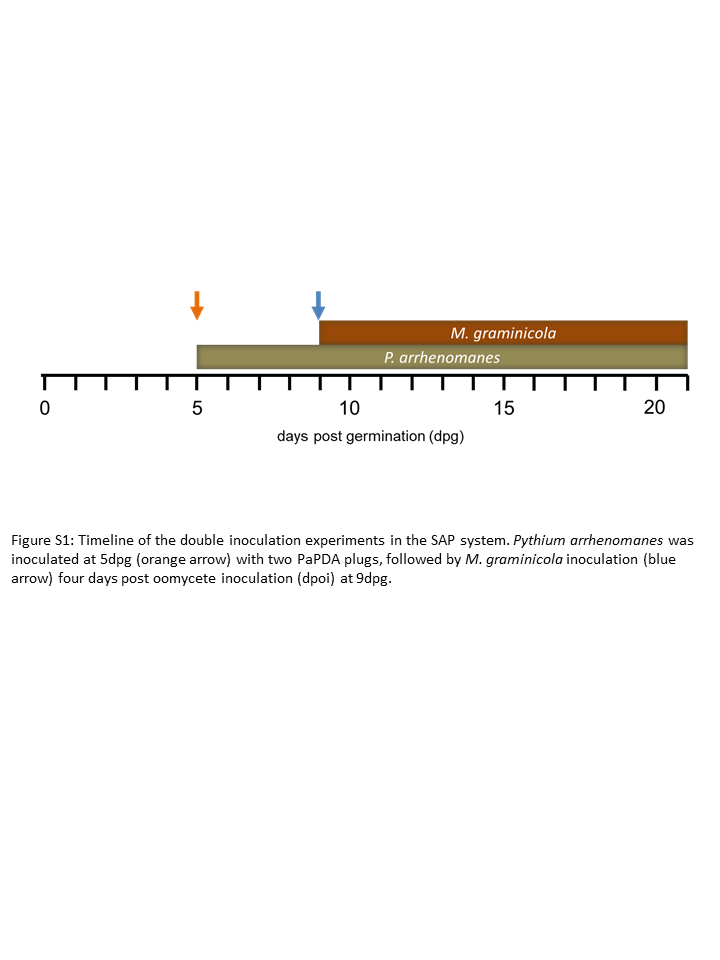

Supplement: Supplementary file 1 [file Image_1.tif]

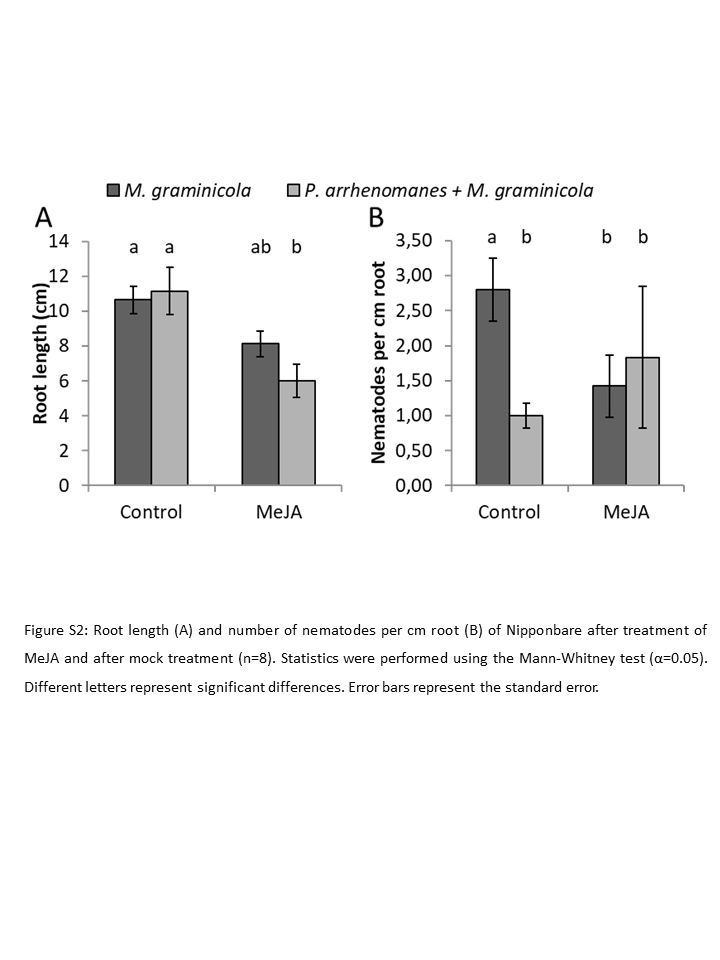

Supplement: Supplementary file 2 [file Image_2.tif]

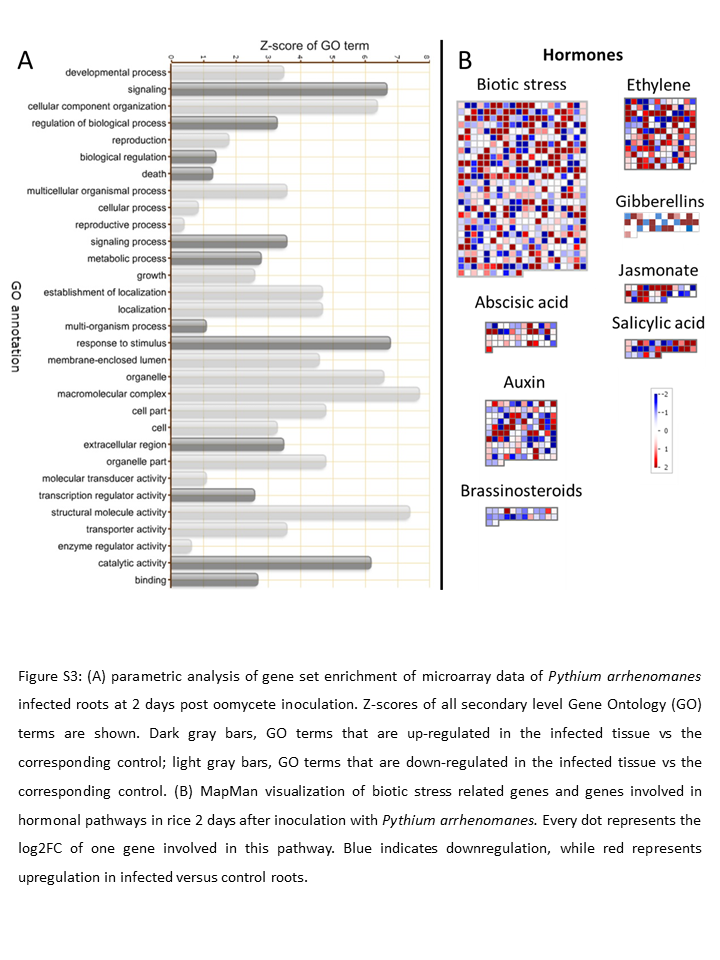

Supplement: Supplementary file 3 [file Image_3.tif]

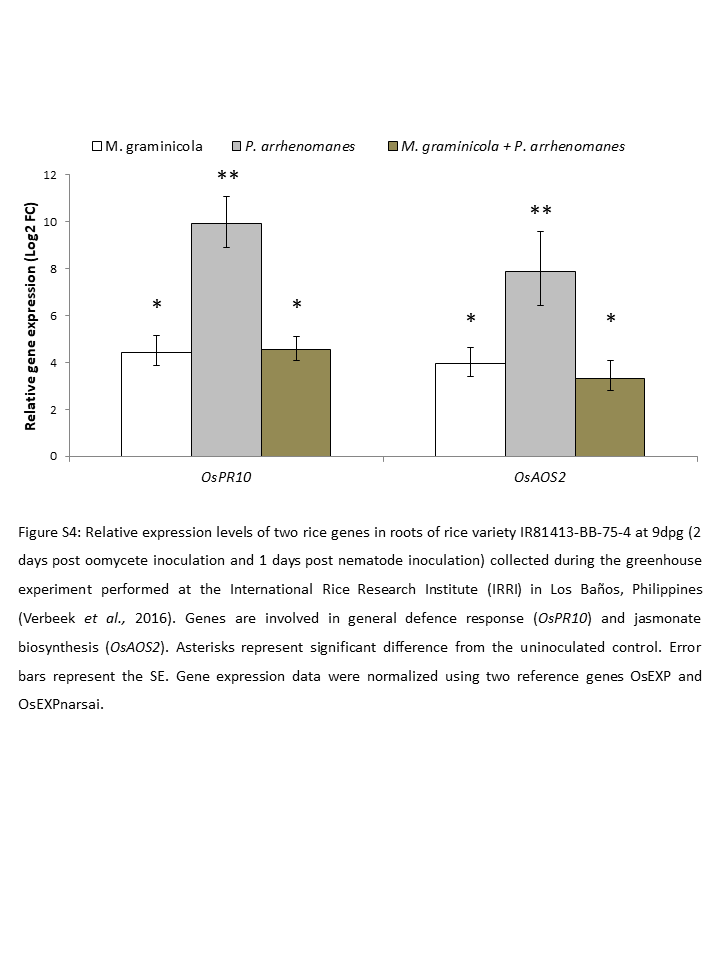

Supplement: Supplementary file 4 [file Image_4.tif]
